# Tudor Domain Containing Protein 3 Promotes Tumorigenesis and Invasive Capacity of Breast Cancer Cells

**DOI:** 10.1038/s41598-017-04955-4

**Published:** 2017-07-11

**Authors:** Alan Morettin, Geneviève Paris, Younes Bouzid, R. Mitchell Baldwin, Theresa J. Falls, John C. Bell, Jocelyn Côté

**Affiliations:** 1 Department of Cellular and Molecular Medicine, University of Ottawa, Ottawa, ON Canada; 2 Faculty of Medicine, University of Ottawa, Ottawa, ON Canada; 3 Center for Innovative Cancer Therapeutics, Ottawa Hospital Research Institute, Ottawa, ON Canada; 4 Department of Biochemistry, Microbiology and Immunology, University of Ottawa, Ottawa, ON Canada

## Abstract

Tudor domain containing protein 3 (TDRD3) is a modular protein identified based on its ability to recognize methylated arginine motifs through its Tudor domain. We have previously shown that TDRD3 localizes to cytoplasmic stress granules, a structure shown to promote survival upon treatment with chemotherapeutic drugs in cancer cells. Here, we report TDRD3 as a novel regulator of cell proliferation and invasion in breast cancer cells. Our study also demonstrates that TDRD3 depletion inhibits tumor formation and metastasis to the lung *in vivo*. Furthermore, we show that TDRD3 regulates the expression of a number of key genes associated with promotion of breast cancer tumorigenesis and disease progression. Strikingly, we report that TDRD3 regulates some of these key targets at the level of translation. These findings provide the first experimental demonstration of a functional role for TDRD3 in promoting breast cancer development and progression, and identify TDRD3 as a potential new therapeutic target for breast cancer.

## Introduction

Similar to all cancers, breast cancer arises through the dysregulation of cellular pathways regulating sensitivity to growth stimuli, cell proliferation and replication, apoptosis, angiogenesis and tissue invasiveness and metastasis^1^. Although therapeutic options are available, in many instances, tumors become refractory to these treatments and the tumor recurs. Therefore, it is crucial to gain a better understanding of how dysregulation of cellular homeostasis contributes to development and progression of breast cancer in order to identify molecular targets for novel treatment options.

Numerous studies have identified dysregulation of arginine methylation as a contributing factor in the development and progression of breast cancer^2, 3^. Accordingly, many of the enzymes that catalyze this post-translational modification, the protein arginine methyltransferases (PRMTs), are over-expressed in breast cancer^4–9^. For example, our group has demonstrated that PRMT7 expression is significantly upregulated in both primary breast tumor tissues and in breast cancer lymph node metastases, identifying PRMT7 as a key player in promoting breast cancer metastatic potential through regulation of MMP9 expression^9^. Similarly, we have previously characterized an alternatively spliced isoform (v2) of PRMT1 that is specifically over-represented in breast cancer and shown that it promotes evasion from apoptosis and metastatic potential in breast cancer cells, at least in part through regulation of β-Catenin expression^10, 11^. Downstream effects of this post-translational modification are mediated by reader/effector proteins containing Tudor domains, which recognize the methylation mark on arginine residue^12–14^.

One such reader/effector protein for arginine methylation is Tudor domain-containing protein 3 (TDRD3). TDRD3 recognizes dimethyl arginine residues generated by PRMT1 and CARM1 on histones^15, 16^, and other proteins^12, 17^. TDRD3 is a modular protein and harbours several other conserved domains in addition to its Tudor domain. Specifically, the N-terminal region contains a DUF/OB fold domain^18, 19^, a poorly characterized domain thought to mediate interactions with nucleic acids, sugars and/or other proteins. Downstream is an ubiquitin binding domain (UBA), which binds Lys_48_-linked tetra-ubiquitin (the signal for proteasome degradation of proteins)^18, 20^. C-terminal to the Tudor domain is the mapped interaction domain with Fragile X Mental Retardation Protein (FMRP)^18, 21, 22^. An exon junction complex binding motif (EBM) follows the FMRP binding domain. The EBM domain allows TDRD3 to interact with various components of early mRNPs, including Y14/Magoh, CBP20/80, and PABP1^21, 23^ (See below). Recognition of histone arginine methylation permits TDRD3 to function as a transcriptional co-activator and regulator of estrogen-mediated gene transcription^15^. In addition, TDRD3 recruits DNA Topoisomerase 3β (TOP3β) to the promoter region of target genes promoting their transcription through resolution of R loop structures^24^. Moreover, TDRD3 serves as a scaffold protein in a trimeric complex with TOP3β and Fragile X Mental Retardation Protein (FMRP)^18, 21, 22, 24^. This complex when associated with messenger ribonucleoproteins (mRNPs) was shown to harbour CBP20/80 but not eIF4E, suggesting that this complex may function in the pioneer round of translation^21^. Furthermore, upon cellular exposure to various environmental stresses, TDRD3 localizes to cytoplasmic stress granules^18, 19^, a structure implicated in cancer cell survival^25^.

## Results

### TDRD3 Regulates Breast Cancer Cells Tumor Growth

**Figure 1:**
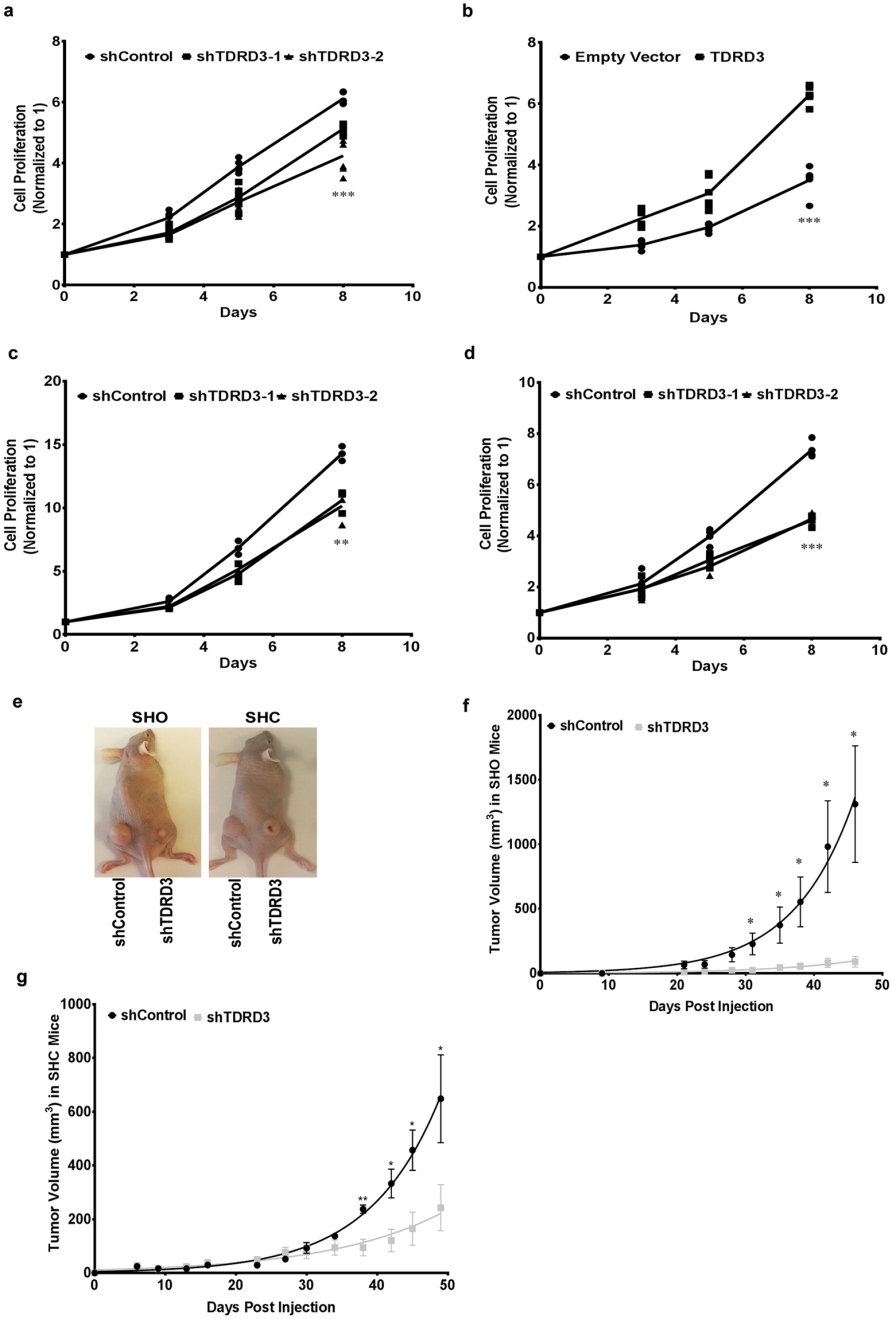
TDRD3 Regulates Cell Proliferation *In Vitro* and *In Vivo*. (**a**) MTT assay were performed on MCF7 shControl (•), shTDRD3-1 (▪), and shTDRD3-2 (▴) cells over a 8 day time course. Data is the mean +/− SEM normalized to 0 h (n = 6, ***p < 0.0001). Significance was determined using a one-way ANOVA. (**b**) MTT assays were performed on MCF7 GFP-tagged empty vector (•) or TDRD3 (▪) cells over a 8 day time course. Data is the mean +/− SEM normalized to 0 h (n = 6, ***p < 0.0001). Significance was determined using a two-tailed *t*-test. MTT assays were performed on (**c**) MDA MB 231 (n = 3, **p = 0.0041) and (**d**) Hs578T (n = 4, ***p < 0.0001) shControl (•), shTDRD3-1 (▪), and shTDRD3-2 (▴) cells over a 8 day time course. Data is the mean +/− SEM normalized to 0 h. Significance was determined using a one-way ANOVA. (**e**) Images of six-week old Crl:SHO-*Prkdc* ^*scid*^ *Hr* ^*hr*^ (SHO) and CB17.Cg-*Prkdc* ^*scid*^ *Hr* ^*hr*^/IcrCrl (SHC) mice injected with MDA MB 231 shControl or shTDRD3 cells in their flank. Tumor volume (mm^3^) of (**f**) SHO (n = 3, Days Post Injection D31 *p = 0.0371) and (**g**) SHC (n = 3, Days Post Injection D38 **p = 0.0070) mice. Tumor volume measurements are the mean +/− SEM. Significance was determined using a two-tailed *t*-test.

It has previously been demonstrated that TDRD3 can act as a co-activator of estrogen-mediated transcription^15^, therefore, we examined whether alteration of TDRD3 expression in estrogen receptor positive (ER^+^) MCF7 breast cancer cells may affect cell proliferation^26^. To investigate this possibility, pools of MCF7 cells expressing a non-targeting (shControl) or a TDRD3-targeting (shTDRD3) shRNA were established (Supplemental Fig. 1a). Using MTT assays to measure cell growth over time, we observed that TDRD3 knockdown resulted in a significant decrease in cell proliferation in two independently selected pools of MCF7 cells (shTDRD3-1, shTDRD3-2) (Fig. 1a). Since TDRD3 knockdown inhibited cell proliferation in MCF7 cells, we next examined whether TDRD3 overexpression could enhance cell proliferation. As predicted, MCF7 cells stably expressing GFP-tagged TDRD3 (Supplemental Fig. 1b) displayed increased proliferation relative to parental cells transfected with an empty vector control (Fig. 1b). Based on these results we next investigated whether TDRD3 could also affect cell proliferation in ER^−^ breast cancer cells. Pools of TDRD3 knockdown cells were established as above (Supplemental Fig. 1c) using MDA MB 231 and Hs578T ER^−^ breast cancer cell lines^27^. In both MDA MB 231 and Hs578T cells, TDRD3 depletion in two independently selected pools of cells (shTDRD3-1, shTDRD3-2) resulted in a significant decrease in cell proliferation, as demonstrated using MTT assays (Fig. 1c and d, respectively). Taken together, these results indicate that TDRD3 can regulate cell growth regardless of estrogen receptor status in breast cancer cells.

To assess whether modulating TDRD3 levels could affect tumor growth *in vivo*, a mouse xenograft model was used. MDA MB 231 cells stably expressing either shControl or shTDRD3 (Supplemental Fig. 1d and e) were subcutaneously injected into the flank of two distinct lines of *scid* mice, Crl:SHO-*Prkdc* ^*scid*^ *Hr* ^*hr*^ (SHO) and CB17.Cg-*Prkdc* ^*scid*^ *Hr* ^*hr*^/IcrCrl (SHC). Injections were performed bi-laterally in these mice to allow for a direct comparison of the growth of these cells in the same mouse. The knockdown of TDRD3 in MDA MB 231 cells resulted in a significant decrease in xenograft tumor growth rate in both mouse models used. In SHO mice, a significant impairment in tumor growth with TDRD3 knockdown was observed at 31 days post injection. At endpoint (46 days), the mean tumor volume observed with TDRD3 knockdown cells was 14.5-fold smaller relative to controls (Fig. 1e and f). Likewise, in the SHC mice, a significant decrease in tumor volume was observed with TDRD3 knockdown beginning at 38 days post-injection. At endpoint, the tumors formed in these mice by the cells expressing shTDRD3 were 2.7-fold smaller than those formed by the control cells (Fig. 1e and g). These results thus demonstrate that TDRD3 is required for efficient growth of breast cancer xenograft tumors.

### TDRD3 Promotes Invasion of Breast Cancer Cells

**Figure 2:**
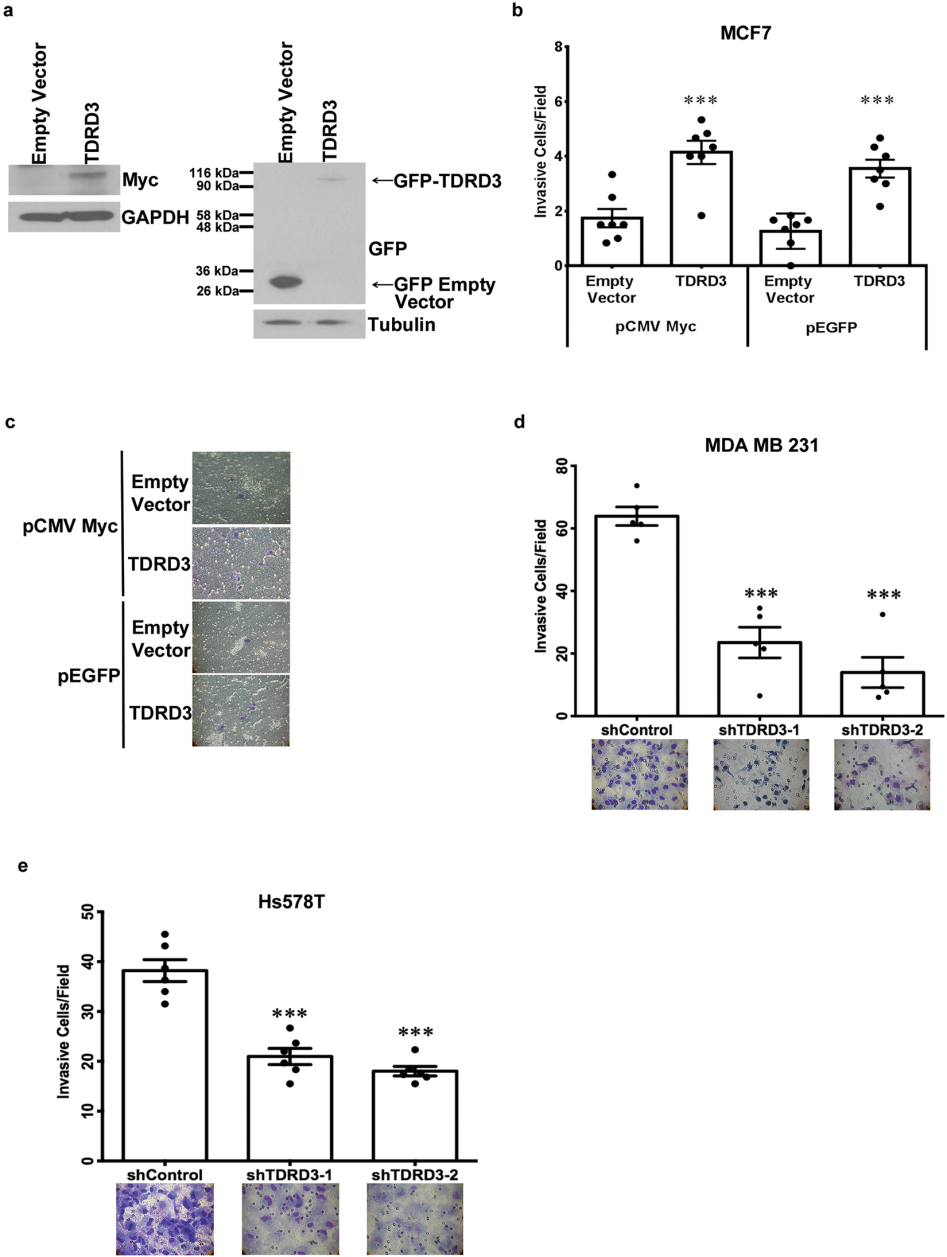
TDRD3 Promotes Cell Invasion in Breast Cancer Cells. (**a**) MCF7 cells were transiently transfected with Myc-tagged TDRD3 or empty vector (left panel) and GFP-tagged TDRD3 or empty vector (right panel) for 48 h and Western blotting was performed (**b**) Invasive cells/field for Myc-tagged TDRD3 or empty vector (n = 7, ***p = 0.0008) and GFP-tagged TDRD3 or empty vector (n = 7, ***p = 0.0001) cells placed in Transwell chambers for 72 h. Data is the mean +/− SEM. Significance was determined using a two-tailed *t*-test. (**c**) Images of Transwell chambers transiently transfected with Myc-tagged TDRD3 or empty vector and GFP-tagged TDRD3 or empty vector (Magnification, 40X). Invasive cells/field of (**d**) MDA MB 231 (n = 5, ***p < 0.0001) or (**e**) Hs578T (n = 6, ***p < 0.0001) shControl, shTDRD3-1 or shTDRD3-2 stable cell lines placed in Transwell chambers for 24 h. Data is the mean +/− SEM. Images of invasive cells (Magnification, 40X) are depicted below graph. Significance was determined using a one-way ANOVA.

The capacity of cancer cells to metastasize to distant sites within the body is dependent on enhanced motility and ability to invade beyond the epithelial basal lamina and extracellular space. In order to determine whether TDRD3 could regulate these hallmarks, Transwell chamber assays were used. First, whether TDRD3 could promote increased motility and invasion in weakly invasive MCF7 cells^26^ was determined. MCF7 cells were transiently transfected with either Myc- (Fig. 2a, left panel) or GFP-tagged (Fig. 2a, right panel) TDRD3 or empty vector. Forty-eight hours post-transfection, cells were plated in motility and invasion chambers for 72 hours. Myc-tagged TDRD3 expression resulted in a 2.4-fold increase in invasion, whereas GFP-tagged TDRD3 promoted a 2.8-fold increase (Fig. 2b and c). Likewise, Myc- and GFP-tagged TDRD3 expression resulted in a 2.1 and 2.8-fold increase in cell motility, respectively (Supplemental Fig. 2a and b). Similar results were obtained using MCF7 cells stably expressing GFP-tagged TDRD3, where overexpression resulted in a 3.4-fold increase in motility (Supplemental Fig. 2c), and a 3.7-fold increase in invasion (Supplemental Fig. 2d). Importantly, these results show that increased TDRD3 expression was sufficient to confer increased motility and invasive properties to intrinsically weakly invasive MCF7 cells.

Next, we sought to determine whether TDRD3 was required to maintain the highly metastatic properties of triple-negative breast cancer cells. Transwell chamber assays were performed using MDA MB 231 and Hs578T cells stably expressing a TDRD3 shRNA. Cells were plated in motility and invasion chambers for 24 hours. In MDA MB 231 cells, TDRD3 depletion (shTDRD3-1, shTDRD3-2) resulted in a 1.4-fold decrease in motility (Supplemental Fig. 2e), and a 2.6 to 4.8-fold decrease in invasion (Fig. 2d). Similarly, in Hs578T cells, TDRD3 knockdown resulted in a 2.1 to 2.3-fold decrease in invasion (Fig. 2e). However, no decrease in motility was observed (Supplemental Fig. 2f).

**Figure 3:**
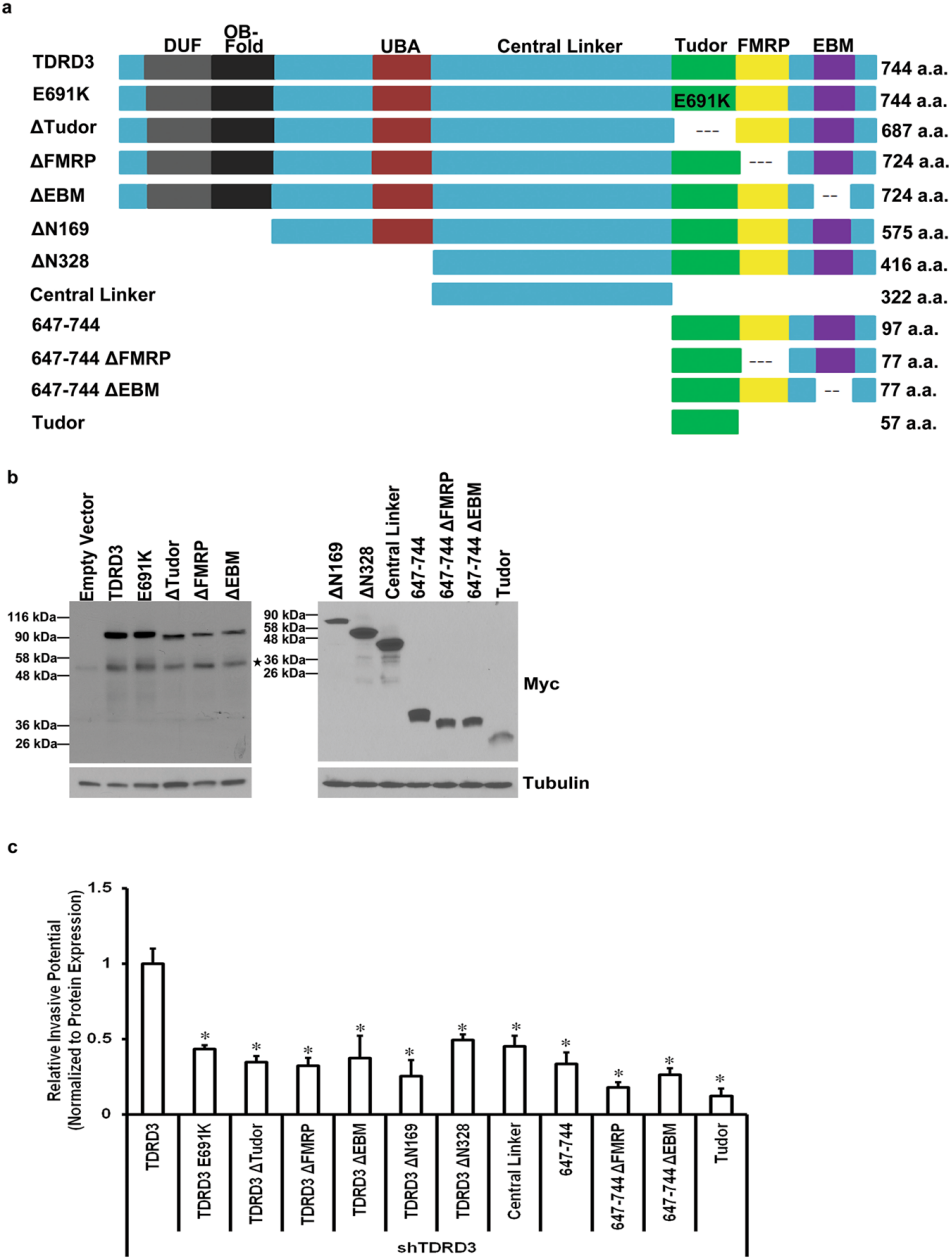
Full Length TDRD3 is Required to Promote Cell Invasion in Breast Cancer Cells. (**a**) Schematic depiction of Myc-tagged TDRD3 wild type or deletion constructs used in rescue experiments. (**b**) Representative Western blot of stably expressing MDA MB 231 shTDRD3 cells transfected with Myc-tagged TDRD3 wild type or deletion mutants for 48 h. Endogenous Myc was detected in some experiments and is indicated by an asterisk. (**c**) Stably expressing MDA MB 231 shTDRD3 cells transfected with Myc-tagged TDRD3 wild type or deletion constructs for 48 h and placed in Transwell chambers for 24 h. Data is the mean +/− SEM (n = 3, *p < 0.05). Data was normalized to protein expression of each construct (n = 3). Myc-tagged TDRD3 full length was set to 1 and the relative invasive potential of each TDRD3 construct was determined. Significance was determined using a one-way ANOVA.

In order to gain mechanistic insight into the function of TDRD3 in promoting breast cancer cell invasion, and to mitigate the possibility of any off-target effects of shRNAs, TDRD3 expression was rescued in MDA MB 231 TDRD3 deficient cells. Specifically, a series of myc-tagged TDRD3 deletion constructs and mutant alleles (Fig. 3a) were transfected into MDA MB 231 shTDRD3 cells for 48 h (Fig. 3b) and Transwell chamber assays were performed after an additional 24 h. As expected, and consistent with a specific effect of TDRD3 in cell invasion, full length myc-TDRD3 was able to fully rescue cell invasion (Fig. 3c). Similarly, invasion was rescued using a GFP-tagged TDRD3 construct (Supplemental Fig. 3). Interestingly, neither the E691K mutant allele, which abrogates TDRD3’s ability to recognize methyl arginines^19^, nor constructs lacking the Tudor domain were able to rescue invasion (Fig. 3c). This insinuates that TDRD3’s ability to recognize methylated arginine residues through its Tudor domain is somehow required for its activity in promoting increased cell invasion. However, the Tudor domain alone was not sufficient to rescue cell invasion. In fact, only the full length wild type construct was able to fully rescue in these experiments (Fig. 3c). Altogether, these results indicate that an intact TDRD3, capable of taking part in interactions with for example TOP3β, components of the EJC, and/or FMRP, is required to promote cell invasion in breast cancer cells. This also suggests that both TDRD3’s known nuclear (transcriptional co-activator) and cytoplasmic (stress granules) roles are potentially part of the mechanism.

### TDRD3 Depletion Inhibits Metastasis of Breast Cancer Cells to the Lungs

**Figure 4:**
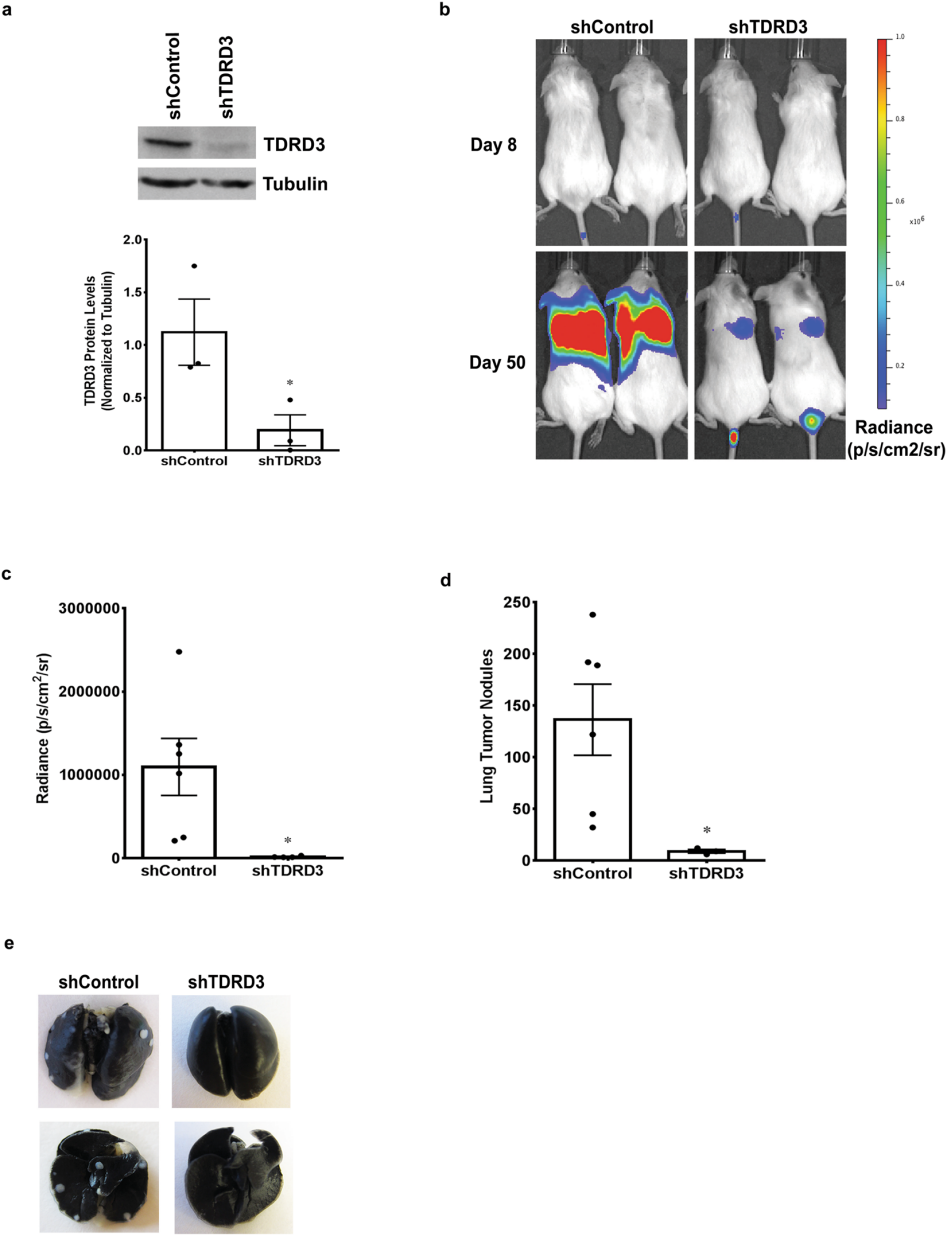
TDRD3 Promotes Metastasis, *In Vivo*. Six-week-old female NOD.CB17-Prkdcscid/JSCID mice were injected with MDA MB 231 shTDRD3 and luciferase or shControl and luciferase expressing cells. (**a**) TDRD3 protein levels, mean ± SEM (n = 3, *p = 0.0274) (**b**) Luciferase intensity was measured using the IVIS spectrum imaging system. Representative images show bioluminescence from shControl and shTDRD3 cells. (**c**) Average radiance (p/s/cm^2^/sr) of shControl (n = 6) and shTDRD3 (n = 4) injected mice (*p = 0.018). (**d**) Lung tumor nodule formation depicting the mean ± SEM for shControl (n = 6) and shTDRD3 (n = 4) mice (*p = 0.0198). (**e**) Images showing the anterior (upper image) and posterior (lower image) lung surface in shControl and shTDRD3 mice. Significance was determined using a two-tailed *t*-test.

To determine if TDRD3 exudes a similar effect on invasion, *in vivo*, a lung metastasis mouse model was employed. This model measures the ability of human cancer cells, which are injected intravenously into the tail vein of immune-compromised mice to migrate through the blood stream, invade the lungs, and form tumour nodules. MDA MB 231 cells stably expressing Control or TDRD3-targeting shRNAs (Fig. 4a) were infected with lentivirus expressing a luciferase cDNA, allowing these cells to be detected using an *in vivo* imaging system (IVIS). Control and shTDRD3 cells were injected into the tail vein of NOD.CB17-Prkdc^SCID^/NrcCrl SCID mice and *in vivo* imaging was performed, eight and fifty days post-injection. No signal was detected for either group at the eight day time-point (Fig. 4b) and this was taken as the basal bioluminescence level. The extent of lung metastasis was then evaluated 50 days post injection and, as expected, the bioluminescent signal (radiance) was localized to the lung area. Importantly, mice injected with TDRD3-depleted cells showed a drastically reduced bioluminescent signal compared to those injected with control shRNA expressing cells (Fig. 4b). As a non-biased approached to measure the metastatic tumor burden within the lungs, the bioluminescent signal for each mouse was quantitated and the mean radiance for each group was determined. This assessment showed that the group injected with TDRD3 knockdown cells had a significantly lower mean radiance compared to the control cells (Fig. 4c), thus indicating a reduction in the metastatic potential of cells *in vivo* with reduced TDRD3 levels. At endpoint (50 days), mice were sacrificed, the lungs were excised and stained with India ink, and the number of tumor nodules determined. Consistent with the bioluminescent data, a significant decrease in lung nodule formation was observed in the lungs of mice injected with shTDRD3 expressing cells (Fig. 4d and e). These results demonstrate that TDRD3 is required to promote breast cancer cell invasion and metastasis to the lungs.

### TDRD3 Regulates Translation in Breast Cancer Cells

**Figure 5:**
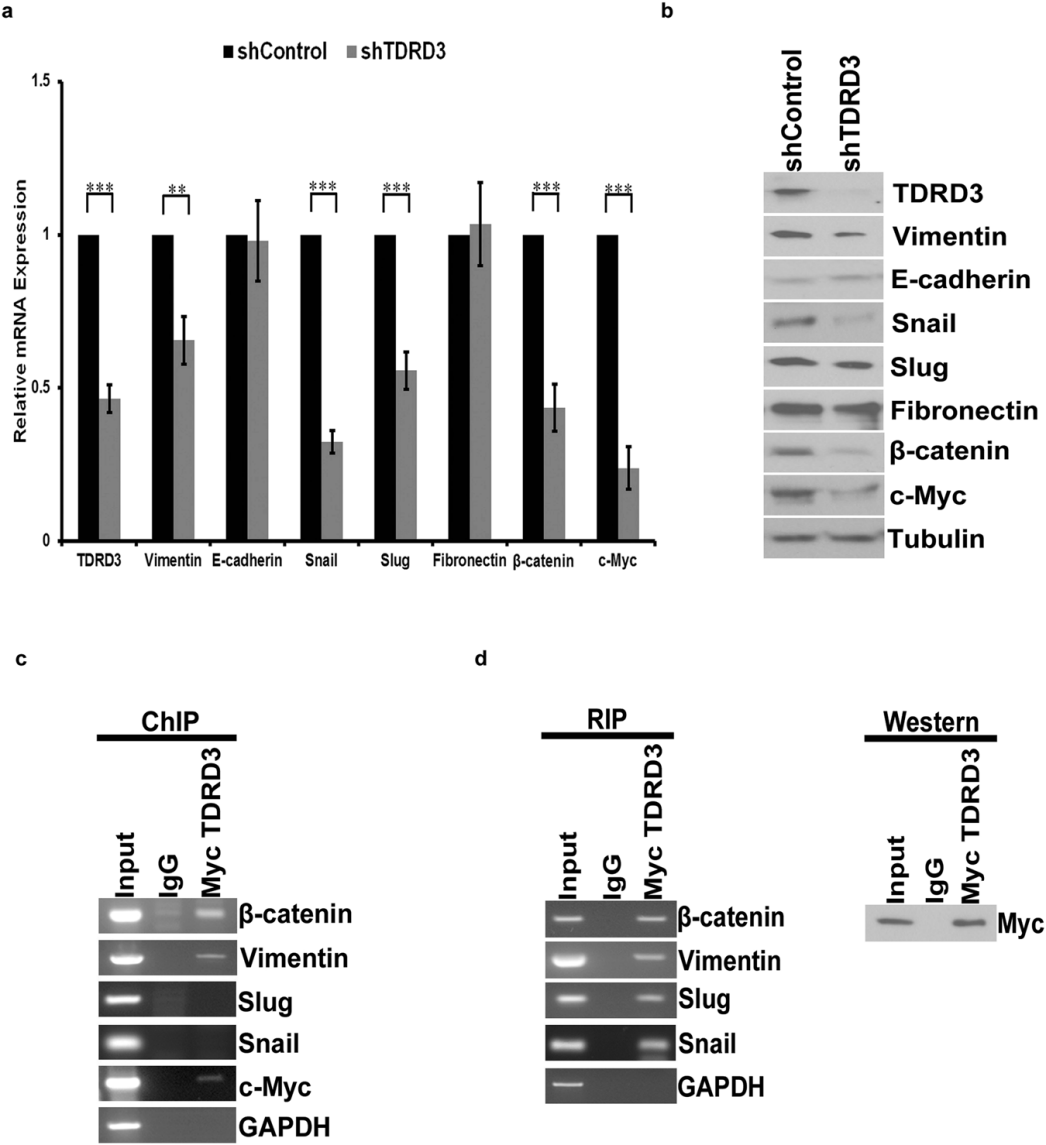
TDRD3 Regulates Epithelial to Mesenchymal Markers in Breast Cancer Cells. (**a**) MDA MB 231 cells were infected with shControl or shTDRD3 expressing lentivirus for 96 h, RNA was isolated, and qPCR was performed from cDNA synthesized from the RNA. Data was normalized to GAPDH and is the mean +/− SEM (n = 6). Significance (**p < 0.01, ***p < 0.001) was determined using a two-tailed *t*-test. (**b**) MDA MB 231 cells were infected with shControl or shTDRD3 expressing lentivirus for 96 h, protein was isolated, and Western blotting was performed. (**c**) Chromatin immunoprecipitation showing MDA MB 231 cells transfected with Myc-tagged TDRD3 for 48 h. (**d**) RNA immunoprecipitation depicting MDA MB 231 transiently transfected with Myc-tagged TDRD3 transfected cells for 48 h (left panel, RNA immunoprecipitation; right panel, Western blot showing myc-tagged TDRD3 immunoprecipitation).

Since altered TDRD3 expression affected cell proliferation and invasion *in vitro*, and tumor growth and metastasis to the lungs *in vivo*, we investigated whether TDRD3 depletion altered the expression of proteins commonly associated with epithelial to mesenchymal transition (EMT) and metastatic potential in breast cancer. MDA MB 231 cells were infected with lentivirus expressing TDRD3 or control shRNAs, cells were harvested and total RNA was isolated. RT-qPCR analysis was performed and revealed a significant decrease in the mRNA levels of MYC proto-oncogene (MYC), a known target of TDRD3^15, 24^, but also for Vimentin (*VIM*), Snail *(SNAI1)*, Slug (*SNAI2*), and β-Catenin (*CTNNB1*) mRNAs (Fig. 5a). Accordingly, TDRD3 knockdown resulted in a decrease in protein expression of *VIM*, *SNAI1*, *SNAI2*, *CTNNB1* and *MYC* (Fig. 5b). In contrast, depletion of TDRD3 had no effect on expression of E-Cadherin *(CDH1)* or Fibronectin *(FN1)* (Fig. 5a and b). These results demonstrate that TDRD3 is a novel regulator of key genes known to be involved EMT and metastasis in breast cancer cells.

To assess whether TDRD3 may regulate these novel targets at the level of transcription, Myc epitope-tagged TDRD3 was exogenously expressed in MDA MB 231 cells, and Myc antibodies were used to immunoprecipitate Myc-TDRD3 from cell lysate and perform chromatin immunoprecipitation (ChIP) analysis. Exogenous TDRD3 expression was performed as efficient and specific immunoprecipitation of endogenous TDRD3 was not achievable in our hands using available antibodies. As above, the previous documented presence of TDRD3 at the *MYC* promoter^15^ was used as a positive control for our ChIP experiments (Fig. 5c). This analysis also revealed enriched occupancy of TDRD3 at the promoters of *CTNNB1* and *VIM* genes (Fig. 5c). In contrast, no enrichment was observed at the promoters of *SNAI2* or *SNAI1*, suggesting that these two targets may be regulated by TDRD3 through a distinct mechanism.

As a first step towards determining whether TDRD3 affected levels of *SNAI2* and/or *SNAI1* through an RNA-related mechanism, we assessed whether TDRD3 associated with these mRNAs, using RNA immunoprecipitation (RIP). Again, MDA MB 231 cells transiently expressing Myc epitope-tagged TDRD3 were used for these experiments (Fig. 5d, right panel). Strikingly, RIP experiments revealed an association of TDRD3 with both *SNAI2* and *SNAI1* mRNAs, but not with *GAPDH* mRNA (Fig. 5d, left panel). Furthermore, TDRD3 was found to also bind *CTNNB1* and *VIM* mRNAs (Fig. 5d, left panel), suggesting that for these targets, TDRD3 may remain associated, directly or indirectly, with the RNA beyond a transcriptional or co-transcriptional step.

**Figure 6:**
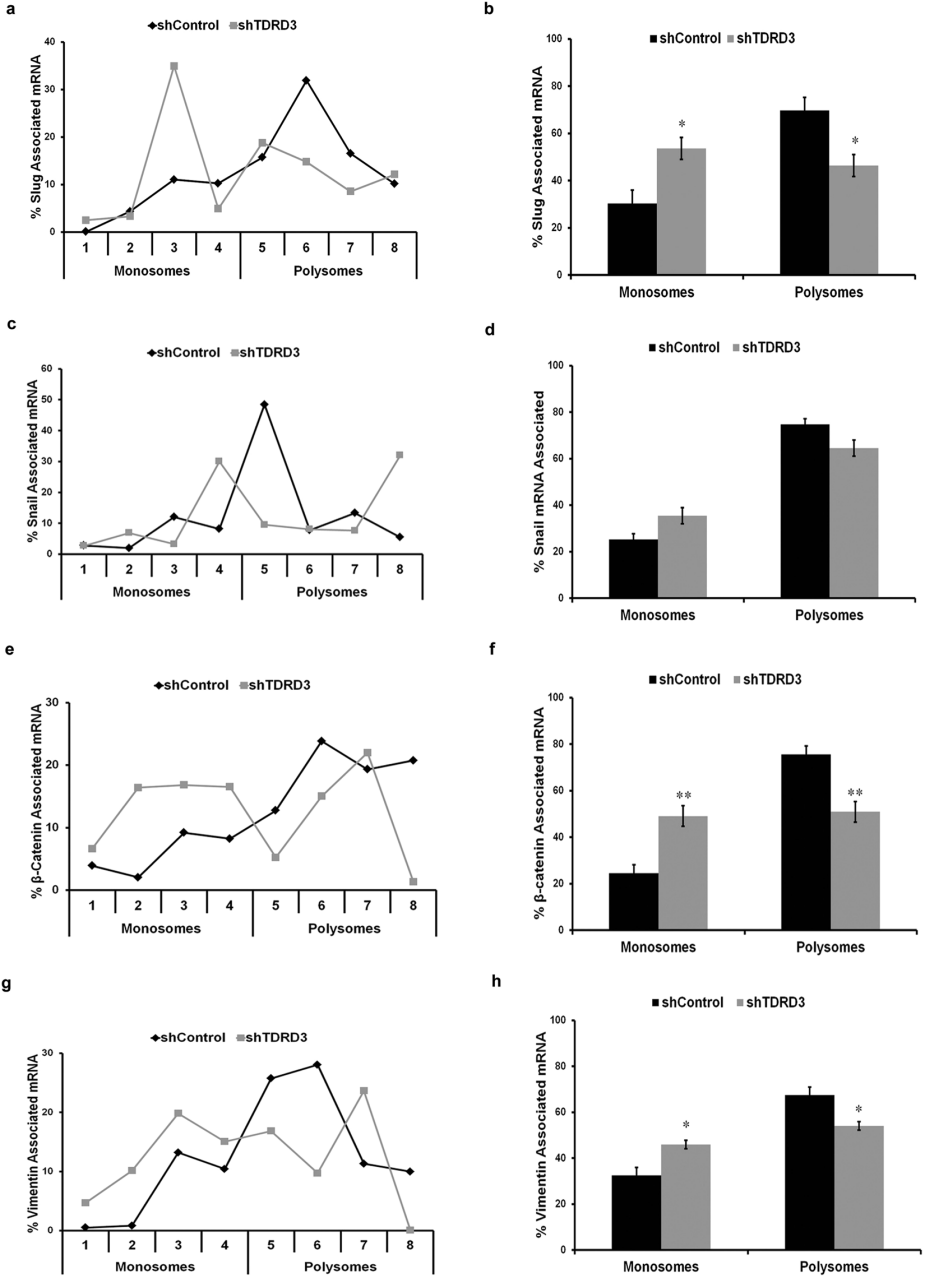
TDRD3 Regulates Translation in Breast Cancer Cells. Cytoplasmic extracts from MDA MB 231 cells infected with shControl or shTDRD3 for 96 h were subjected to fractionation on a 10–45% sucrose gradient. RNA was isolated from each fraction and RT-qPCR analysis was performed determining (**a,b**) Slug, (**c,d**) Snail, (**e,f**) β-catenin, and (**g,h**) Vimentin mRNA distribution. The line graph is a representative result of one independent trial, while the bar graph represents the pooled mRNA association from each monosome and polysome fraction. Significance (n = 4, *p < 0.05, **p < 0.01) was determined using a two-tailed *t*-test.

We have been the first to report that TDRD3 associates with polyribosomes and can be found in cytoplasmic stress granules^19^, findings that have since been corroborated by other groups^18–22, 28^. Nevertheless, a direct role for TDRD3 in translation has not been demonstrated. Based on our results with *Slug* and *Snail*, we investigated whether TDRD3 may regulate the expression of these mRNAs at the translational level. MDA MB 231 cells were infected with lentivirus expressing either a TDRD3 or control shRNA and post-nuclear supernatants were subjected to sucrose gradient sedimentation (Supplemental Fig. 4a), essentially as we have previously reported^19, 29^. RNA was isolated from monosome and polysome fractions and relative mRNA distribution determined by RT-qPCR. Strikingly, TDRD3 depletion resulted in a drastic shift in *SLUG* mRNA distribution, from predominantly heavy polysomal to monosome fractions (Fig. 6a), suggesting that TDRD3 is required for efficient translation of *SNAI2 * mRNA. This result was confirmed by plotting the mean *SNAI2* mRNA distribution from four independent experiments, using pooled monosomal vs polysomal fractions (Fig. 6b). A similar tendency was observed for *SNAI1* mRNA, although statistical significance was not quite achieved (p = 0.0518; Fig. 6c,d). Interestingly, statistically significant shifts from polysomal to monosome fractions were observed for *CTNNB1* and *VIM* mRNA in TDRD3 depleted MDA MB 231 cells (Fig. 6e–h, respectively). In contrast, no difference in polysome profile distribution was observed for *MYC* mRNA between control and shTDRD3 MDA MB 231 cells (Supplemental Fig. 4b,c). Taken together, our results show that TDRD3 can selectively promote translation of a specific subset of mRNAs in breast cancer cells.

## Discussion

We have documented in the current study a novel role for TDRD3 in promoting tumorigenesis and metastasis in breast cancer cells. Specifically, dysregulation of TDRD3 expression altered cell proliferation in culture, with depletion impeding tumor xenografts growth in mice. In highly aggressive breast cancer cells, knockdown resulted in decreased cell invasion and reduced lung metastasis, while in weakly metastatic cells, overexpression of TDRD3 was sufficient to confer to them the ability to invade through an extracellular-like matrix. We show that TDRD3 promotes the expression of a number of genes known to be crucial players in the EMT process in breast cancer cells, including *SNAI1*, *SNAI2*, *CTNNB1* and *VIM*. Lastly, we demonstrate, for the first time, that TDRD3 regulates the expression of at least some of these genes at the level of translation.

In previous studies, TDRD3 was shown to act as a transcriptional co-activator at estrogen receptor dependent promoters^15^. While MCF7 breast cancer cells were used for those experiments, the potential influence of TDRD3 on the cancer-related features of these cells was never explored. We report here that TDRD3 promoted efficient cell growth in ER^+^ MCF7 cells, but importantly, it also exerted this effect in two different ER^−^ breast cancer cell lines. Moreover, TDRD3 depletion in these cells drastically impeded tumor growth in mouse xenograft experiments. This suggests that TDRD3 may regulate cell growth through more than one mechanism. Interestingly, TDRD3 is enriched at the *MYC* promoter, functioning as a co-activator of MYC-mediated gene transcription, and promotes the recruitment of a topoisomerase (TOP3β) to chromatin in order to prevent R loop accumulation^15^. As previously reported^24^, *Myc* mRNA expression was reduced upon TDRD3 knockdown in MCF7 cells, a phenotype also observed here in MDA MB 231 cells. Since increased MYC expression is oncogenic and a driver of uncontrolled cell proliferation in many cancers^30–33^ including breast cancer, it is conceivable that TDRD3 may regulate breast cancer cell proliferation and tumor growth at least in part through this pathway. Intriguingly, TDRD3 is also a transcriptional target of MYC^34^, suggesting that it may function as part of a feed-forward mechanism sustaining high Myc expression. In future experiments, it would be interesting to explore the potential beneficial effect of targeting and reducing TDRD3 expression in cancers known to be ‘addicted’ to MYC over-expression.

Our current study has also uncovered a role for TDRD3 in promoting increased cell motility and invasion *in vitro*, and metastasis to the lung in an experimental metastasis model *in vivo*. Again, these effects were observed in both ER^+^ luminal cells (MCF7) as well as triple negative cells (e.g. MDA MB 231). Intriguingly, however, TDRD3 knockdown did not affect cell motility in Hs578T cells. This suggests that TDRD3 may regulate motility through more than one molecular mechanism and/or could reflect intrinsic differences in the signaling pathways active in each of these cell lines, although they are both of the Claudin-low subtype. Our demonstration that TDRD3 promotes high metastatic potential may explain, at least in part, the fact that it was identified as a top hit in two separate unbiased genomic studies searching for gene expression signatures correlating with poor prognosis in late stage disease of triple-negative breast cancers^35, 36^.

Using a candidate approach, we have identified in the current study a number of novel target genes regulated by TDRD3, including *β-Catenin*, *Vimentin*, *Snail*, and *Slug*. β-Catenin expression is enriched in highly aggressive, metastatic breast tumors and is associated with an overall decrease in patient survival^37^. Furthermore, its expression is necessary for cell migration and invasion in highly aggressive breast cancer cell lines^38^. Vimentin is a component of intermediate filaments and its involvement in cell adhesion, migration, and invasion is well characterized^39, 40^. Increased vimentin expression in cancer cells is also indicative of a mesenchymal cell phenotype^41^. In breast epithelial cells, the transcription factors SNAIL and SLUG are key central regulators of an epithelial to mesenchymal transition program promoting metastasis, drug resistance, and stem cell traits. Although *CDH1* is a known transcriptional target downstream of SNAIL and SLUG^42, 43^, we have not observed any increase in *CDH1* mRNA or protein expression. This may be due to the documented methylation occurring on the promoter of *CDH1* in MDA MB 231 cells, thus inhibiting *E-CADHERIN* expression^44^. Alternatively, TDRD3 may regulate CDH1 by affecting its localization at the plasma membrane, a mechanism also known to take place during EMT. We have detected TDRD3, using ChIP, in the promoter region of *CTNNB1* and *VIM*. Interestingly, the *VIM* locus is known to undergo R-loop formation, with resolution promoting its active transcription^45^, although the mechanism by which R-loop resolution is mediated was not determined. It is thus a possibility that TDRD3 may recruit TOP3β to its locus in order to resolve R-loop formation and promotes its efficient transcription. However, to date, evidence of R loop formation on the promoter of *CTNNB1* is lacking. Finally, we cannot exclude that TDRD3 may also promote cell invasion and metastasis through its regulation of *MYC* transcription as described above.

Although our results strongly suggest that TDRD3 regulates *CTNNB1* and *VIM* expression at the transcriptional level, we have uncovered that it also promotes their efficient translation, as well as that of *SNAI2*. For *SNAI1*, an effect of reduced TDRD3 expression on its translation was not as pronounced, and did not quite reach statistical significance. Since TDRD3 was not found at the *SNAI1* promoter, but was present in association with *SNAI1* mRNA, TDRD3 may regulate this target at the level of mRNA stability, although this would require further investigation. A cytoplasmic role for TDRD3 was first proposed by us and Utz Fischer’s group in 2008 when it was shown to be a novel protein re-localizing to so-called ‘stress granules’ upon cell exposure to various environmental stresses^18, 19^. These foci are tightly coupled to translational regulation and are thought to represent sites of mRNA triage influencing post-transcriptional gene expression programs through selective translation and mRNA stability^46^. Stress granules have also been linked with cancer, potentially contributing to tumor survival and chemoresistance^25^, so it would be interesting in future studies to investigate whether re-location of TDRD3 to these structures is part of its role in breast cancer. In addition to its presence within stress granules, TDRD3 was also found to associate with polyribosomes^18, 19, 21, 22, 28^. Although this previous work led to the proposition that TDRD3 may somehow participate in translational regulation, our current study provides the first experimental demonstration that TDRD3 can regulate translation of specific mRNAs.

TDRD3 is thought to exist in cells mostly as part of a trimeric protein complex, with TOP3β and Fragile X Mental Retardation Protein (FMRP)^21, 22, 24^. The functional relevance of this trimeric complex has so far been studied mostly in the context of neuronal cells, where it was shown to contribute to an expression program important for synapse formation and normal neurodevelopment and mental health^21, 22^. TDRD3 was postulated to function as a scaffolding protein in this trimeric complex. Our *in vitro* invasion rescue experiments indeed demonstrated a requirement for the N-terminal portion of TDRD3 (ΔN169), which mediates its interaction with TOP3β^21, 22, 24^, and the FMRP-interacting motif (ΔFMRP)^18^ to promote cell invasion. Interestingly, FMRP was recently shown to promote breast cancer cell invasion and lung metastasis through regulation of specific mRNAs involved in EMT, including *Vimentin* mRNA^47^. However, in contrast to our results, *E-Cadherin* was amongst the mRNAs that were regulated by FMRP^47^. While FMRP has been implicated in breast cancer progression, to date, a role for TOP3β has yet to be documented. Thus, our results suggest that TDRD3’s function within the trimeric complex with TOP3β and FMRP may be target-specific. Whether TDRD3’s function in breast cancer cell invasion and metastasis is dependent on its involvement in the trimeric complex will require further experimentation.

Our results show that TDRD3’s ability to recognize dimethyl arginine residues is required to rescue efficient breast cancer cell invasive potential as ΔTudor or E691K mutant alleles were not able to rescue invasion. Since the Tudor domain is involved in mediating arginine methylation-dependent interactions^12, 15–17, 19^, this suggests that one or more PRMT(s) may be implicated upstream of TDRD3 in breast cancer cells. Interestingly, PRMT1 and CARM1, for which TDRD3 is thought to act as a downstream reader/effector molecule^15, 17, 24^, have both been shown to promote various hallmarks of breast cancer^11, 48–50^. Of particular interest is our previous work showing that a predominantly cytoplasmic isoform of PRMT1, PRMT1v2, specifically promotes metastatic potential and survival of breast cancer cells^10, 11^. So, it will be important in future studies to explore whether TDRD3 and specific PRMTs act in the same pathway(s) to promote breast cancer development and disease progression.

## Materials and Methods

### Cell Lines and Transfections

Cell lines were purchased from the American Type Culture Collection. 293T and MDA MB 231 cells were cultured in Dulbecco’s Modified Eagles Medium supplemented with 2 mM glutamine and 10% fetal bovine serum. Hs578T and MCF7 cells were supplemented with 2.75 µg/ml insulin. Transfections were performed using Lipofectamine 2000 (Life Technologies) according to the manufacturer’s protocol. The pGIPz TDRD3 shRNA plasmid (RHS4430-200228777) was purchased from Dharmacon. Lentivirus was produced in 293T cells using the lentiviral packaging vectors pMDG.2 and psPAX2. Stably expressing shTDRD3 cell lines were established by selection with 2 µg/mL puromycin. Stably expressing GFP-tagged TDRD3 cells were established by selection with 500 µg/mL G418.

### Antibodies

TDRD3 antibodies used were from Abnova (H00081550-BO1P) and Bethyl Laboratories (A302-692A). The Tubulin antibody was purchased from Sigma Aldrich (T6199) and the β-catenin antibody from EMD Millipore (06-734). The Slug antibody was purchased from Cell Signalling Technology (9585), E-cadherin from BD Bioscience (610181), Fibronectin (ab2413) and Snail (ab17732) from Abcam, and c-Myc from Santa Cruz (sc-789). A murine hybridoma secreting an anti-myc monoclonal antibody (9E10) was purchased from ATCC (CRL-1729). The Vimentin antibody was purchased from BD Biosciences (550513), the GFP antibody from Chromotek (3H9) and the GAPDH antibody from BioLegend (MMS-580S).

### DNA Constructs

pCMV Myc TDRD3 expression constructs (TDRD3, E691K, ΔN328, Central Linker, 647-744) were previously described^19^. The pCMV Myc Tudor construct was created through PCR amplification of the Tudor domain, inserting the product into the *EcoRI/XhoI* restriction sites. ΔEBM constructs were generated through restriction digest of TDRD3 with the *MscI* restriction enzyme. *MscI* cleaves TDRD3 internally before the EBM domain. ΔFMRP constructs were generated through mutagenesis with primers flanking the FMRP binding domain: 5′-atcaagcccattcaaggccagccaagacga-3′ and 5′-tcgtcttggctggccttgaatgggcttgat-3′. The ΔTudor construct was created using primer sets that generated PCR products skipping the Tudor domain P1: 5′-caggaaaagggtccctcctttgcagaggc-3′ and 5′-ctcccatgcctctgtcttgttgtcttccca-3′ and P2: 5′-tgggaagacaacaagacagaggcatgggag-3′ and 5′-acctcccacacctccccctgaacc-3′. The PCR products were digested with AanI and ligated back together. TDRD3 was cloned into the pEGFP-C1 expression vector at the *EcoRI/BamHI* restriction sites. All constructs were verified by sequencing.

### MTT Assay

MTT assays were performed as previously described^11^. Absorbance was read at 590 nm using a BIOTEK Synergy H1 Multi-Mode Plate Reader.

### Cell Motility and Invasion Assays

Those experiments were performed essentially as we have described previously^11^. For Transwell chamber assays, 24-well BD control or Biocoat Matrigel invasion chambers containing 8-micron pores (BD Biosciences) were used to assess cell motility and invasion, respectively, according to the manufacturer’s specifications. Briefly, 50,000 cells in serum-free DMEM were seeded into each chamber and placed in a 24-well plate containing 500 μL complete DMEM and incubated for 24 h or 72 h. A minimum of four random fields at 20x magnification were counted to determine the number of cells that passed through the chamber membranes.

### *In Vivo* Mouse Experiments

Experiments were conducted with the approval of the University of Ottawa Animal Care Committee and Animal Care and Veterinary Services according to the Canadian Council on Animal Care guidelines.

### Xenograft Mice Experiments

Experiments were performed on five week old female CB17.Cg-*Prkdc* ^*scid*^ *Hr* ^*hr*^/IcrCrl (SHC) and Crl:SHO-*Prkdc* ^*scid*^ *Hr* ^*hr*^ (SHO) (Charles River, Montreal, Qc, Canada). SHC (n = 3) or SHO (n = 3) mice were subcutaneously injected on opposite flanks with MDA MB 231 cells stably expressing a non-targeting (shControl) or TDRD3-targeting (shTDRD3) shRNA (10 million cells/200 µL PBS). Tumor diameter was measured twice per week using a digital caliper (Marathon Watch Company Ltd, Richmond Hill, ON, Canada). Experiments were terminated when tumor diameter reached 1.7 cm (around 50 days post-injection). Tumor volumes were determined using the formula:

$${\rm{Volume}}\,{\rm{of}}\,{\rm{tumor}}\,{\rm{in}}\,{{\rm{mm}}}^{3}=({\rm{Length}}\times {{\rm{Width}}}^{2})/2,\,{\rm{where}}\,{\rm{Length}}={\rm{longest}}\,{\rm{measurement}}.$$

### Tail Vein Injections

Six-week-old female NOD.CB17-Prkdc^scid^/JSCID mice (Charles River, Montreal, QC, Canada) were injected intravenously in the tail vein with MDA MB 231 cells stably expressing luciferase and shControl or luciferase and shTDRD3 (500 000 cells/100 µL PBS). Imaging and analysis of lung tumor nodule formation were performed as previously described^9^.

### Immunoblotting

Immunoblotting were performed as previously described^51^.

### RT-qPCR

RNA extraction and cDNA synthesis was performed as previously described^51^. RT-qPCR was performed in a CFX384 Touch qPCR (Bio-Rad) using IQ^TM^ Sybr Green Supermix (Bio-Rad). PCR reactions were performed in triplicate with data acquisition and analysis performed using Bio-Rad CFX Manager data software. GAPDH was used as an endogenous reference gene. Primer pairs are listed in Supplemental Table S1.

### Sucrose Gradient Fractionation

MDA MB 231 cells were infected with shControl or shTDRD3 expressing lentivirus for 96 hours. Sucrose gradient fractionation was performed as previously described^29^. RNA was isolated from fractions and percent mRNA association was determined by qPCR using the delta delta (Ct) value. Primer pairs are listed in Supplemental Table S1.

### Chromatin Immunoprecipitation

DNA: protein complexes were cross-linked using 0.75% formaldehyde for 10min. Reactions were quenched with 125 mM glycine for 5 min, and cells were lysed using FA Lysis Buffer (50 mM HEPES-KOH pH 7.5, 140 mM NaCl, 1 mM EDTA pH 8.0, 1% Triton X-100, 0.1% Sodium Deoxycholate, 0.1% SDS). DNA was sonicated using a Covaris S220 Ultra-Sonicator and immunoprecipitation reactions were performed overnight using 25 µg. Samples were incubated for 2 h with protein A/G agarose beads (pre-blocked with 75 ng/µl beads of salmon sperm DNA and 100 ng/µl beads of BSA). Beads were washed sequentially using wash buffer (0.1% SDS, 1% Triton X-100, 2 mM EDTA, 150 mM NaCl, 20 mM Tris pH 8.0), final wash buffer (0.1% SDS, 1% Triton X-100, 2 mM EDTA pH 8.0, 500 mM NaCl, 20 mM Tris pH 8.0), and LiCl wash buffer (0.25 M LiCl, 1% NP-40, 1% Sodium Deoxycholate, 1 mM EDTA, 10 mM Tris pH 8.0). Samples were eluted from beads with Elution Buffer (1% SDS, 100 mM NaHCO^3^) for 15 min at 30 °C, reverse cross-linked overnight at 65 °C, and incubated with 20 µg RNase A and 40 µg proteinase K for 1 h at 45 °C. DNA was purified using a PCR Purification Kit (Qiagen). Primer pairs are listed in Supplemental Table S1.

### RNA Immunoprecipitation

RNA immunoprecipitations reactions were performed as previously described^52^. Primer pairs are listed in Supplemental Table S1.

### Statistical Analysis

Two-tailed *t*-tests or one-way analysis of variance (ANOVA) were used to determine statistical significance. A minimum of three independent biological replicates was performed for each experiment.

## Electronic supplementary material

Supplemental Information
