# Supplementary material for: Tudor Domain Containing Protein 3 Promotes Tumorigenesis and Invasive Capacity of Breast Cancer Cells
Source: Sci Rep. 2017 Jul 11;7:5153. doi: 10.1038/s41598-017-04955-4 (PMC5506013; doi:10.1038/s41598-017-04955-4)
Supplement: Supplementary file 1 — Supplemental Information [file 41598_2017_4955_MOESM1_ESM.pdf]

Supplemental Information (SI): Supplemental Figures and Tables

Tudor Domain Containing Protein 3, Promotes Tumorigenesis and Invasive Capacity of Breast Cancer Cells

Alan Morettin[1,2], Genevieve Paris[1,2], Younes Bouzid[1,2], R. Mitchell Baldwin[1,2], Theresa J. Falls[3], John C. Bell[3,4], Jocelyn Côté[1,2]*

[1] Department of Cellular and Molecular Medicine, University of Ottawa, Ottawa, ON, Canada

[2] Faculty of Medicine, University of Ottawa, Ottawa, ON Canada

[3] Center for Innovative Cancer Therapeutics, Ottawa Hospital Research Institute, Ottawa, ON, Canada

[4] Department of Biochemistry, Microbiology and Immunology, University of Ottawa, Ottawa, ON, Canada

Table of Contents

Supplemental Figures…………………………………………………………2

Supplemental Table S1………………………………………………………10

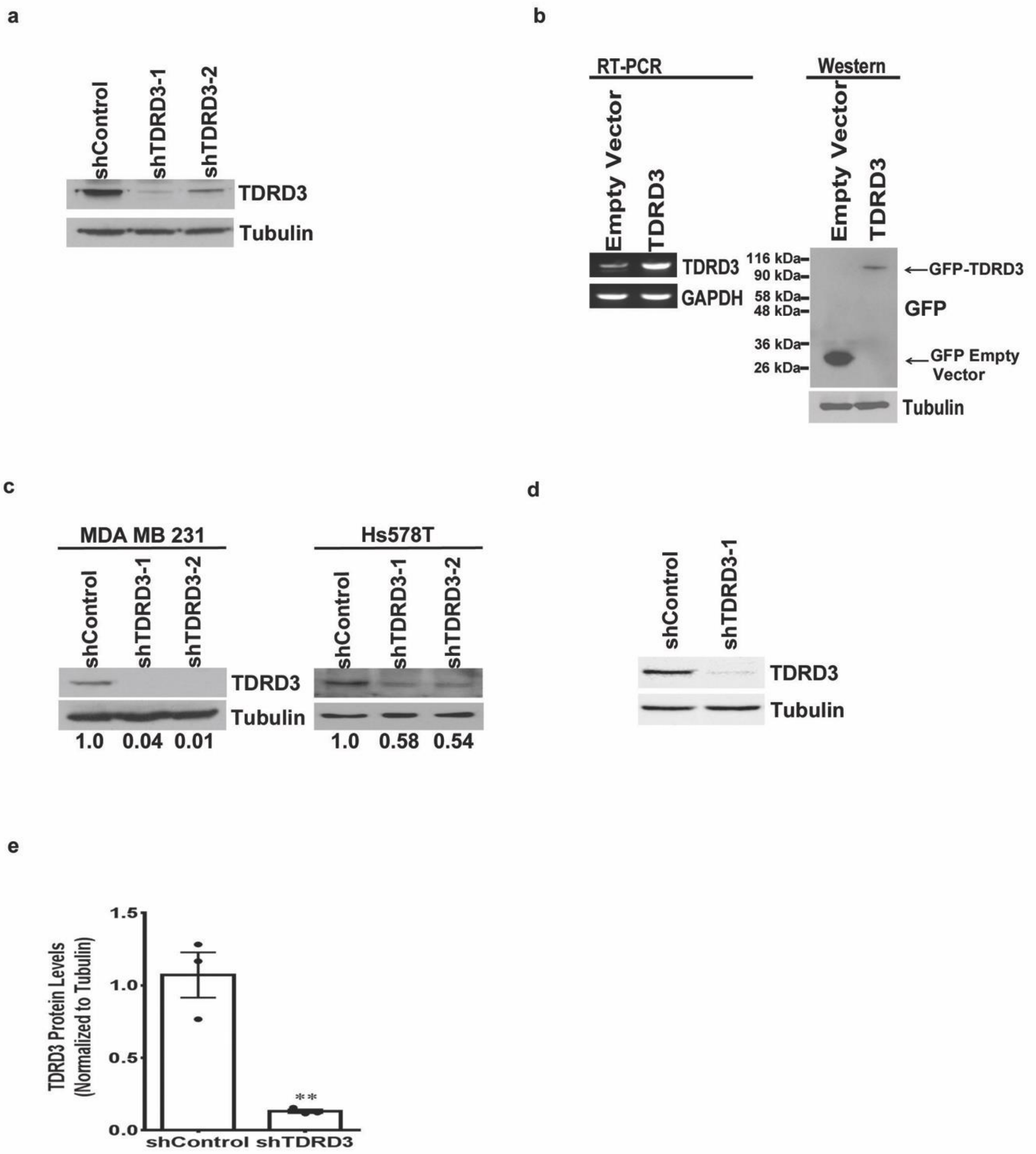

a
shControl
shTDRD3-1
shTDRD3-2
TDRD3
Tubulin
b
RT-PCR
Empty Vector
TDRD3
TDRD3
GAPDH
Western
Empty Vector
TDRD3
116 kDa
90 kDa
58 kDa
48 kDa
36 kDa
26 kDa
GFP-TDRD3
GFP
GFP Empty Vector
Tubulin
c
MDA MB 231
shControl
shTDRD3-1
shTDRD3-2
TDRD3
Tubulin
1.0 0.04 0.01
Hs578T
shControl
shTDRD3-1
shTDRD3-2
1.0 0.58 0.54
d
shControl
shTDRD3-1
TDRD3
Tubulin
e
TDRD3 Protein Levels (Normalized to Tubulin)
1.5
1.0
0.5
0.0
**
shControl
shTDRD3

Supplemental Figure 1: TDRD3 Promotes Cell Proliferation in Breast Cancer Cells

(a) Western blotting depicting MCF7 cells stably expressing a control non-targeting shRNA (shControl) or a TDRD3-targeting shRNA (shTDRD3). (b) RT-PCR (left panel) and Western blotting (right panel) showing stably expressing GFP-tagged TDRD3 or empty vector MCF7 cells lines. (c) Western blotting showing MDA MB 231 (left panel) or Hs578T (right panel) cells stably expressing shControl or shTDRD3. TDRD3 expression normalized to Tubulin is depicted below the Western Blot. (d) Western blot analysis depicting TDRD3 protein expression in MDA MB 231 shControl or shTDRD3 cells injected into the flank of SHO and SHC mice (n=3). (e) Densitometry analysis of TDRD3 protein expression of the mean ± SEM (n=3, $^{**}p=0.0039$).

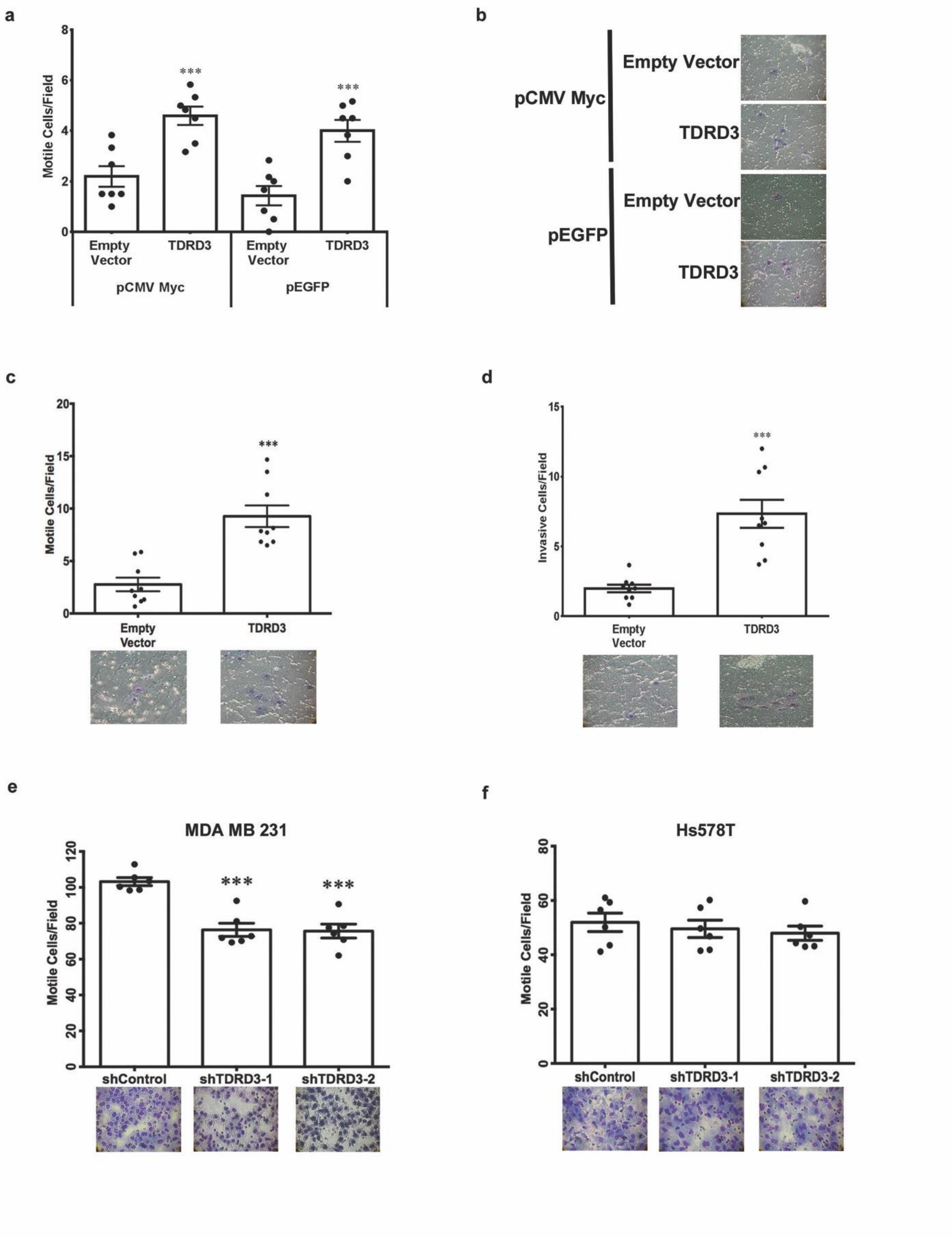
a
Motile Cells/Field
***
Empty Vector
TDRD3
pCMV Myc
pEGFP
b
pCMV Myc
Empty Vector
TDRD3
pEGFP
Empty Vector
TDRD3
c
Motile Cells/Field
***
Empty Vector
TDRD3
d
Invasive Cells/Field
***
Empty Vector
TDRD3
e
MDA MB 231
Motile Cells/Field
***
shControl
shTDRD3-1
shTDRD3-2
f
Hs578T
Motile Cells/Field
shControl
shTDRD3-1
shTDRD3-2

Supplemental Figure 2: TDRD3 Promotes Increased Cell Motility and Invasion in Breast Cancer Cells

(a) Motile cells/field of MCF7 cells transiently transfected with Myc-tagged TDRD3 (n=7, ***p=0.0009) or empty vector and GFP-tagged TDRD3 (n=7, ***p=0.0008) or empty vector incubated in Transwell chambers for 72h. Data is the mean +/- SEM. (b) Representative images depicting motile cells/field of MCF7 cells transiently transfected with Myc-tagged TDRD3 or empty vector and GFP-tagged TDRD3 or empty vector (Magnification, 40X). (c) Motile cells/field (n=9, ***p<0.0001) or (d) Invasive cells/field (n=9, ***p<0.0001) of stably expressing MCF7 GFP-tagged TDRD3 or empty vector incubated in Transwell chambers for 72h. Data is the mean +/- SEM. Significance was determined using a two-tailed *t*-test. Representative images are shown below the graphs (Magnification, 40X). (e) Motile cells/field of MDA MB 231 (n=6, ***p=0.0001) or (f) Hs578T (n=6) shControl, shTDRD3-1 or shTDRD3-2 stably expressing cells incubated in Transwell chambers for 24h. Data is the mean +/- SEM. Significance was determined using a one-way ANOVA. Representative images of motile cells/field in MDA MB 231 and Hs578T shControl and shTDRD3 cells are depicted below the graph (Magnification, 40X).

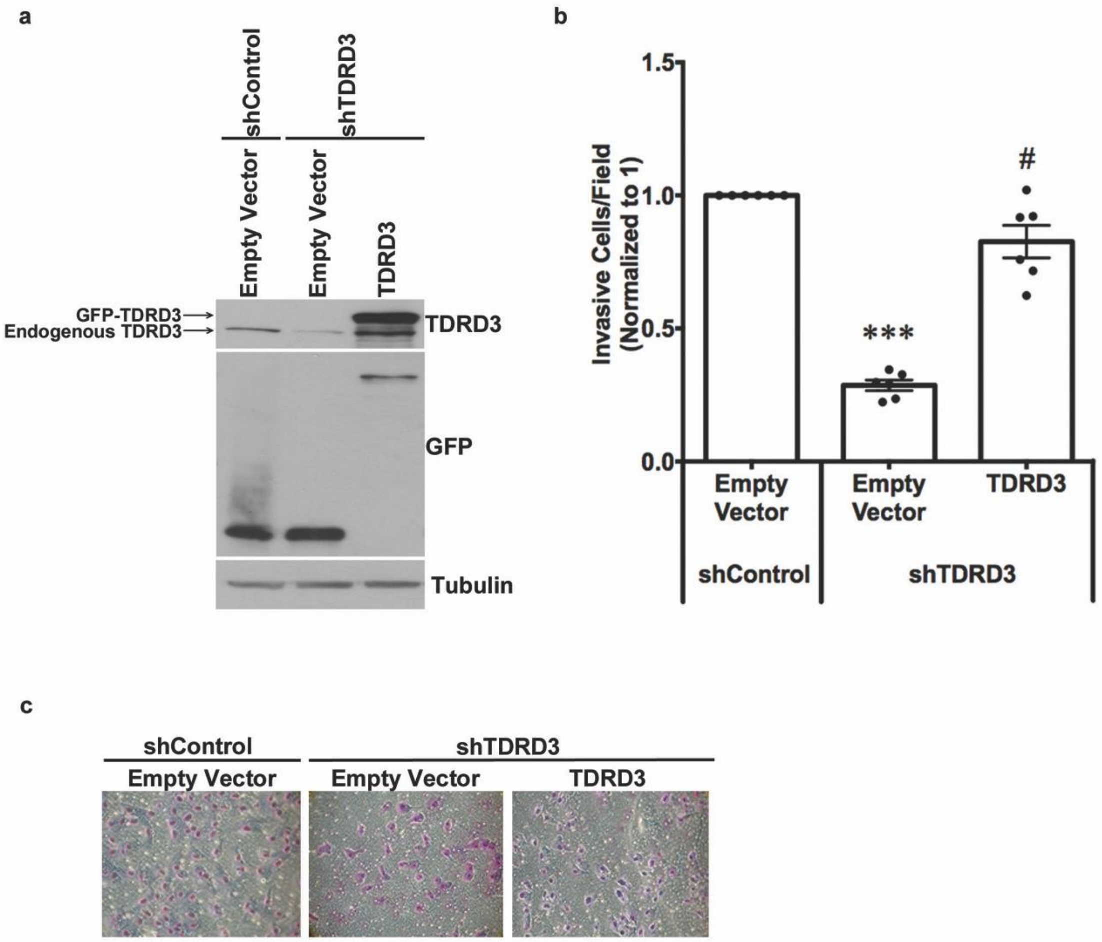
a
shControl
shTDRD3
Empty Vector
Empty Vector
TDRD3
GFP-TDRD3
Endogenous TDRD3
TDRD3
GFP
Tubulin
b
Invasive Cells/Field (Normalized to 1)
1.5
1.0
0.5
0.0
***
#
Empty Vector
Empty Vector
TDRD3
shControl
shTDRD3
c
shControl
shTDRD3
Empty Vector
Empty Vector
TDRD3

Supplemental Figure 3: TDRD3 Rescues Cell Invasion in TDRD3 Depleted Cells

(a) MDA MB 231 TDRD3 shRNA cells were transfected with GFP-tagged TDRD3 or empty vector for 48h and then incubated in invasion chambers for 24h. (b) Invasive cells/field depicting the mean +/- SEM. Significance was determined as compared to shControl Empty Vector (n=6, ***p<0.0001) or to shTDRD3 Empty Vector (n=6, #p=0.0019) determined using a two-tailed *t*-test (c) Images of invasive cells/field (Magnification, 40X).

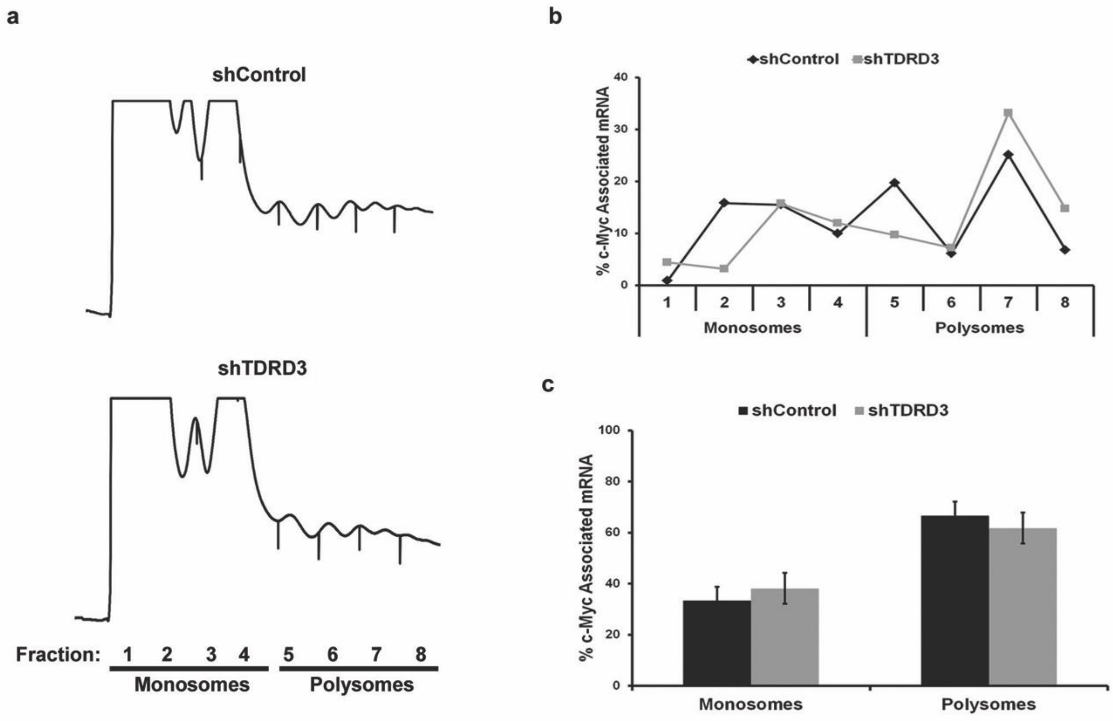
a
shControl
shTDRD3
Fraction: 1 2 3 4 5 6 7 8
Monosomes
Polysomes
b
shControl
shTDRD3
% c-Myc Associated mRNA
40
30
20
10
0
1 2 3 4 5 6 7 8
Monosomes
Polysomes
c
shControl
shTDRD3
% c-Myc Associated mRNA
100
80
60
40
20
0
Monosomes
Polysomes

Supplemental Figure 4: TDRD3 Regulates Translation in Breast Cancer Cells

MDA MB 231 cells were infected with lentivirus expressing shControl or shTDRD3 for 96h, and cytoplasmic extracts were subjected to fractionation on a 10-45% sucrose gradient. (a) Representative polysome profiles from MDA MB 231 shControl (top panel) and shTDRD3 (bottom panel) lentivirus expressing cells. (b, c) RNA was isolated from each sucrose fraction in MDA MB 231 shControl or shTDRD3 expressing cells and qPCR was performed examining the mRNA distribution. Statistical significance (n=4, *$p<0.05$) determined using a two-tailed *t*-test.

Supplemental Table S1: Primer Sets

Gene Name	Forward	Reverse
qPCR/RIP Primers		
TDRD3	CCAGGCGGGTTGGTATCTTT	ACACATGGACCTTCGAGCTT
Snail	CGAGTGGTTCTTCTGCGCTA	CTGCTGGAAGGTAAACTCTGGA
Vimentin	GACAATGCGTCTCTGGCACGTCTT	TCCTCCGCCTCCTGCAGGTTCTT
GAPDH	ACCACAGTCCATGCCATCAC	TCCACCACCCTGTTGCTGTA
c-Myc	TTCGGGTAGTGGAAAACCAG	AGCAGCTCGAATTTCTTCCA
Fibronectin	GCACCAACTGACCTGAAG	GCCACCATAAGTCCTGATAC
E-cadherin	TGGGTTATTCCTCCCATCAG	TTTGTCAGGGAGCTCAGGAT
Slug	TGTTGCAGTGAGGGCAAGAA	GACCCTGGTTGCTTCAAGGA
B-catenin	AAAATGGCAGTGCGTTTAG	TTTGAAGGCAGTCTGTCGTA
ChIP Primers		
Vimentin	CGCTAGGTCCCTATTGGCTG	GGTGGACGTAGTCACGTAGC
B-catenin	GCGCCATTTTAAGCCTCTCG	TAAGGAAAGGAGCGCCCAAG
Slug	GTTCACAGCTGTCCCAGAGG	ATGTGTGTCCAGTTCGCTGT
Snail	GTGCGTTTCCCTCGTCAATG	GGACACCTGACCTTCCGAC
c-Myc	GATGCGGTTTGTCAAACAGTACT	GAGATTAGCGAGAGAGGATCTT
GAPDH	TCCTCCTGTTTCATCCAAGC	AGTAGCCGGGCCCTACTTT
